# Barriers and facilitators to retaining a cohort of street-based cisgender female sex workers recruited in Baltimore, Maryland, USA: results from the SAPPHIRE study

**DOI:** 10.1186/s12889-020-08723-4

**Published:** 2020-04-29

**Authors:** Bradley E. Silberzahn, Miles B. Morris, Katelyn E. Riegger, Rebecca Hamilton White, Catherine A. Tomko, Ju Nyeong Park, Katherine H.A. Footer, Steven S. Huettner, Susan G. Sherman

**Affiliations:** 1 Johns Hopkins Bloomberg School of Public Health, Department of Health, Behavior & Society, 624 N Broadway, Baltimore, MD 21205 USA; 2 Department of Pediatrics, Johns Hopkins School of Medicine, 5200 Eastern Avenue, Baltimore, MD 21224 USA

**Keywords:** Sex work, Female sex worker, Retention, Sexually transmitted infection, Human immunodeficiency virus

## Abstract

**Background:**

Despite experiencing HIV/STIs, violence, and other morbidities at higher rates than the general public, street-based female sex workers are often absent from public health research and surveillance due to the difficulty and high costs associated with engagement and retention. The current study builds on existing literature by examining barriers and facilitators of retaining a street-based cohort of cisgender female sex workers recruited in a mobile setting in Baltimore, Maryland who participated in the SAPPHIRE study. Participants completed interviews and sexual health testing at baseline, 3-, 6-, 9-, and 12-months.

**Methods:**

Retention strategies are described and discussed in light of their benefits and challenges. Strategies included collecting several forms of participant contact information, maintaining an extensive field presence by data collectors, conducting social media outreach and public record searches, and providing cash and non-cash incentives. We also calculated raw and adjusted retention proportions at each follow-up period. Lastly, baseline sample characteristics were compared by number of completed visits across demographic, structural vulnerabilities, work environment, and substance use variables using F-tests and Pearson’s chi-square tests.

**Results:**

Although there were drawbacks to each retention strategy, each method was useful in tandem in achieving a successful follow-up rate. While direct forms of contact such as phone calls, social media outreach, and email were useful for retaining more stable participants, less stable participants required extensive field-based efforts such as home and site visits that increase the likelihood of random encounters. Overall, adjusted retention exceeded 70% for the duration of the 12-month study. Participants who were younger, recently experienced homelessness, and injected drugs daily were less likely to have completed all or most follow-up visits.

**Conclusion:**

Retention of street-based female sex workers required the simultaneous use of diverse retention strategies that were tailored to participant characteristics. With familiarity of the dynamic nature of the study population characteristics, resources can be appropriately allocated to strategies most likely to result in successful retention.

## Background

Female sex workers (FSW), people who use drugs (PWUD), and people experiencing homelessness are disproportionately affected by HIV/STIs, violence, overdose, and other morbidities at higher rates than their similarly aged peers [1–8]. These individuals are sometimes “hidden” to researchers and therefore underrepresented in relevant public health research and surveillance, given the intensive costs and difficulty associated with engagement [9–13]. The myriad of challenges associated with researchers and service providers engaging high-risk populations has led to their designation as “hard-to-reach” [9]. While conventional methods of recruitment in research studies may not be practical for hard-to-reach populations, structural vulnerabilities can also challenge their retention in longitudinal research studies.

Poor retention can lead to significant differences between participants who complete study follow-up visits and those who do not and threatens statistical power, both of which ultimately reduce a study’s validity and generalizability [14, 15]. These biases can lead to a lack of understanding of hard-to-reach populations who are often in the greatest need. Given the importance of their inclusion, researchers have examined the barriers and facilitators associated with retaining these populations [9]. Maintaining contact with participants has consistently been identified as a primary barrier, necessitating multiple strategies to bolster retention. Strategies include: building rapport through a range of mechanisms; offering incentives (cash and non-cash) or gifts for study participation; distributing transportation vouchers; branded study items; obtaining several means of contact (e.g., phone numbers, social media accounts, multiple addresses, and stable contacts); and conducting home visits [9, 16–29].

Although a significant body of research exists, literature on retaining hard-to-reach populations is limited with most studies focusing on fixed locations or utilizing postal or web-based participation, while also omitting high risk populations such as FSW [9]. Retention of participants recruited in a mobile setting presents unique challenges due to the absence of a permanent location for them to contact or visit without prompting. Fixed-sites have several advantages, primary of which being travel to such a location is somewhat of an initial screener, increasing the likelihood that those who come to the site return for future visits. The most vulnerable are also less likely to be able to travel to fixed-sites, resulting in further underrepresentation in fixed-site studies.

Currently, the few studies examining retention techniques targeting sex workers generally focus on broad sex worker samples (e.g., males, females) or are they are a subset of other populations (e.g., PWUD) [9]. Sex worker characteristics and experiences vary widely by venue of employment (e.g., street- and venue-based, online), gender, and the legal status of sex work in the jurisdictions in which they work [30]. Depending on the setting, beneficial methods of retention may differ widely. While retaining venue-based sex workers may only require phone calls or occasional site visits, retaining more transient street-based populations can necessitate costly, extensive field-based outreach. Street-based cisgender FSW (CSFW) in the US are often impacted by several overlapping and reinforcing structural vulnerabilities such as homelessness, incarceration, and a history of injection drug use that in the context of research, challenge retention techniques given lack of stable housing and reliable forms of contact [31–34]. Their under representation in research can have a real impact on receipt of funding and relevant programs targeted to their unique health needs.

The current study examines barriers and facilitators to retaining a street-based CFSW cohort recruited in a mobile setting in Baltimore, Maryland. Specifically, we aim to provide a detailed description and discussion of the follow-up strategies used to retain street-based CFSW as well as analyze follow-up rates and demographic differences between study participants who were and were not lost to follow-up. We conclude with a discussion of the successes and shortcomings of our strategies in relation to the broader retention literature to provide suggestions for future research.

## Methods

### The SAPPHIRE study

The Sex Workers And Police Promoting Health In Risky Environments (SAPPHIRE) study was a prospective longitudinal cohort study that examined the role of police in shaping the HIV and STI risk environment of street-based FSW [2, 32, 35–37]. From April 2016 to August 2017, 250 CFSW were recruited through targeted sampling from 11 street-based locations in Baltimore using a mobile research van. Sixty-two transgender female sex workers (TFSW) also completed the SAPPHIRE study, however they are not included in this analysis due to differences in retention strategies (i.e., use of a peer navigator, reliable forms of communication). The sampling methods have been previously detailed elsewhere [35]. The mobile research van used was a 30-ft-long recreational vehicle (RV) configured with two private interview booths and a restroom for participants to self-collect biological specimens.

Women were eligible to participate in the study if they met the follow criteria: (1) age ≥ 15 years old (2); sold or traded oral, vaginal or anal sex for money or “things like food, drugs, or favors;” (3) picked up clients on the street or in public places at least 3 times within the past 3 months (4); willing to undergo HIV and STI testing. Exclusion criterion were: (1) identifying as male or a man (2); being unwilling or unable to provide contact information to be reached for future visits. Written consent was obtained from all interested and eligible participants. Participants who were under the age of 18 received individualized health counseling with study supervisors, which included having a detailed conversation on service needs and referrals to known providers.

The SAPPHIRE cohort was followed from baseline through four subsequent follow-up visits at 3-, 6-, 9-, and 12-months. Participants had a 2-month window to complete each follow-up (2 weeks prior through 6 weeks after their interview date) and were permitted to complete follow-ups regardless of whether they had completed previous visits. At each visit, participants completed an interviewer-administered Computer Assisted Personal Interview (CAPI) survey and were tested for HIV, gonorrhea, trichomonas, and chlamydia. Participants who relocated more than 1 h away from Baltimore were permitted to complete interviews by phone; however, no biological specimens were collected. A community advisory board (CAB) comprised of current and former FSW provided insight and suggestions for all study procedures. The study was approved by the Johns Hopkins Bloomberg School of Public Health Institutional Review Board. Data are unavailable due to privacy concerns for participants.

### Participant characteristics

SAPPHIRE participants were an average of 36 years old (range: 18–61 years), 66% were non-Hispanic White, 23% non-Hispanic Black, and 11% were Hispanic or other race or ethnicity. The sample was characterized by several structural vulnerabilities. At baseline, 62% reported recently experiencing (past 3 months) homelessness, 74% reported daily non-injection drug use, 58% reported injecting drugs daily, 54% reported going to sleep hungry at least once per week, and 47% reported having been arrested in the past year.

### Retention strategies

We employed several population-specific strategies to maximize the potential for follow-up encounters through the duration of the study. Study management prioritized collecting several forms of participant contact information, maintaining an extensive field presence by data collectors, conducting social media outreach and public record searches, and providing cash and non-cash incentives.

#### Locator forms

At each study visit, participants completed a standard locator form. Locator form fields included: participant name; physical description; primary phone number; email and social media accounts; addresses; three locations frequented by participants; and phone numbers and addresses of two stable contacts. “Stable contacts” were defined as anyone with whom participants had communicated with in the past 3 months. Participants were required to provide either one direct form of contact (e.g., phone or social media) and at least one stable contact. If participants were unable to provide a direct form of contact, then two stable contacts were required. Participants who were unable to provide either one direct form of contact and a stable contact or two stable contacts were prohibited from enrolling in the study. When communicating with anyone other than participants (contacts or people who answered participants’ primary number), study staff referred to the study as a “women’s health study” to protect participant confidentiality. Given the high prevalence of injection drug use among our study population [38–40], study staff also recorded whether participants attended the Baltimore Syringe Services Program (SSP) and if so, which SSP locations they visited.

#### Scheduling follow-up appointments

Two weeks prior to the beginning of a participant’s eligibility window, staff made phone calls, sent text messages, emails, and private social media messages to notify participants of upcoming study visit and van locations and times. A study phone and laptop were kept in the study office and on the study van for staff use. Study staff were permitted to send private messages regarding eligibility and scheduling to participants using social media with SAPPHIRE Study *Facebook* and *Instagram* accounts. When primary forms of contact failed, study staff attempted to contact the participant’s stable contacts to relay messages about upcoming van shifts. As follow-up van shifts approached, in-office study staff continued contact attempts. All eligibility and contact attempt information was documented and stored electronically using Research Electronic Data Capture (REDCap) tools hosted at Johns Hopkins University [41, 42]*.*

#### Mobile van shifts

Follow-up van schedules were created monthly, and shifts lasted 4 h. Locations were chosen based on the greatest number of eligible participants. Since recruitment and follow-up occurred simultaneously, 1–2 shifts a week were designated for follow-up interviews to ensure that there was available space on the van to accommodate all study participants. Once the entire cohort had been recruited, 3–5 follow-up shifts were scheduled per week, depending on the number of eligible participants. Van shift times varied based on the initial targeted sampling framework [35].

When the van arrived at a zone, staff canvassed the area to locate study participants. Study staff would approach women and inquire about potential follow-up eligibility by describing the study van and study procedures, referring to the study only as a women’s health study. If someone encountered was thought to be a participant, they were brought to the van to check their enrollment and follow-up window. If an individual was enrolled, eligible for follow-up, and interested in completing their interview, study staff would complete a new contact form and continue with the remainder of the follow-up visit. Staff also updated contact information if participants were not eligible. After surveying the area for potential participants, staff returned to the van to contact all eligible participants recruited from that zone.

#### Participant tracking

Participants who did not complete a follow-up interview within a month and a half of eligibility were assigned to a designated “tracking” team who attempted to locate participants through targeted street outreach during the remaining 2 weeks of their follow-up window. Tracking teams traveled in pairs in personal vehicles to all addresses listed on the participant’s locator form to find the participant. As many participants reported drug injection, tracking staff also visited Baltimore SSP locations during the times participants provided on their locator form. Like van shifts, if participants were not eligible, the tracking team updated contact information in REDCap.

#### Maryland judiciary case search

Staff also used a public web-based database, Maryland Judiciary Case Search (Case Search) [43], to learn whether participants were currently incarcerated and therefore not available for follow-up. Case Search provided information on all civil, traffic, and criminal cases. Listed information included defendant name, address, case number, date of birth, trial date, charge, case disposition, and sentencing information. Study staff used listed information to determine participant availability for follow-up and to verify addresses for tracking.

#### Incentives

SAPPHIRE participants received $70 USD prepaid VISA debit cards for baseline and 12-month visits, and $45 USD prepaid VISA debit cards for the 3-, 6-, and 9-month visits. Study staff also distributed non-monetary incentives including condoms, naloxone, lip balm, hand sanitizer, and cleansing wipes. All incentives except condoms and naloxone were labeled with the SAPPHIRE study logo and phone number. Study management ensured tracking teams and the study van were fully stocked with supplies, beverages, and candy. With input from the CAB, study management chose these specific non-cash incentives due to their practicality with the target population (e.g., injection drug use, homelessness). Participants were provided with non-monetary incentives at each encounter regardless of eligibility.

#### Staff composition & rapport Building

The SAPPHIRE field staff team was comprised of a continuous group of 10–15 diverse (e.g., gender, age, sexual orientation, race/ethnicity) full- and part-time employees with varying backgrounds and decades of combined, relevant experience. Study staff brought an array of expertise in the field with some having extensive public health research experience, clinical/nursing experience, or experience in HIV/STI linkage to care with the Baltimore City Health Department. Study management held routine meetings with field staff to obtain feedback on study protocols.

Study management hosted several staff trainings to ensure staff operated in a manner that made participants feel safe, comfortable, and at ease during all study procedures and interactions. Staff underwent periodic trainings that focused on harm reduction for FSW and PWUD, provided a framework for the factors that placed the study populations at risk, and contextualized the criminalized nature of sex work and drug use in Baltimore: Considerations for Working with Cisgender and Transgender Female Sex Workers; Drug Use 101; Supporting Survivors and Staff in Research on Violence; Harm Reduction 101; Data Collection Protocols and Staff Safety; Racial Justice; and Baltimore City Resource Referrals. Prepared with this understanding and a diverse set of life experiences, staff established trust and ongoing relationships with study participants. When possible, the same staff were assigned to track participants at subsequent visits to further contribute to rapport building.

The in-person interview format used in this study often led to larger conversations between participants and interviewers outside of the specific survey questions. Extensive neighborhood and need-specific (e.g., housing, health care, drug treatment, food) resource guides were developed to help connect participants to service providers following the visit. At the request of participants, staff assisted in linking them to qualified organizations to ensure that the needs of the participant were met.

### Analysis of follow-up rates

#### Outcomes

Retention rates were analyzed to understand the impact of the range of strategies that were employed. Retention was defined as having successfully completed a follow-up visit within the two-month window. For study staff retention efforts, we calculated a raw retention proportion and an adjusted retention proportion, removing participants who missed a follow-up visit for any of the following reasons during the study: incarceration, death, relocation from Baltimore, enrollment in in-patient drug treatment, refusal to participate in the study, and removal from the study. Both raw and adjusted retention proportions were calculated at each follow-up period. Calculations were based on the number of participants that completed their follow-up study visit at each period divided by the total study sample, minus those who met one of the six above listed circumstances in the adjusted retention proportion calculations. The adjusted retention proportion helped guide and motivate study staff efforts because these situations circumstantially prevented participants from being located or interviewed, and thus, attention was shifted to participants who could possibly be reached to complete their next survey. Participants could miss individual study visits and remain in the study, reentering at any future follow-up time point.

A secondary outcome was the total number of visits participants completed out of the five study visits. For this outcome, we compared participants’ baseline characteristics by the total number of visits they completed. To be considered as a completed study visit, the study visit had to have been completed within the allotted two-month eligibility window. The possible range for number of completed visits was 1 to 5.

#### Independent variables

Age was retained as a continuous covariate. Race/ethnicity was trichotomized into non-Hispanic White, non-Hispanic Black, or other. We explored: relationship status (single vs. married, in a relationship); number of financial dependents (≥1 dependents vs. none); children less than 18 years old living with participants (yes vs. no); limited education (high school/GED graduate or higher vs. less than high school graduate); homelessness (yes vs. no); arrest in the past 12 months (yes vs. no) and food insecurity (going to bed hungry ≥1 per week). Childhood (< 18 years) abuse was defined as ever being pressured or forced into sexual intercourse or sexual touching, or being hit, punched, slapped or otherwise physically hurt by someone causing marks or physical injury. Work environment variables were: engagement in sex work daily; 30 or more clients in the past 3 months; length of time in street-based sex work (≤5 years vs. 6+ years); other locations where clients were found included indoor environments (e.g., clubs, bars), online, or via referrals from either other clients or sex workers. We also asked about substance use, dichotomizing into daily or less than daily non-injection or injection drug use. Marijuana use was not considered for the daily non-injection drug use variable.

#### Analytical sample and statistical analysis

The sample was comprised of 250 CFSW. Women were recruited and retained through the methods described above. We compared baseline sample characteristics by number of completed visits across demographic, structural vulnerabilities, work environment, and substance use variables using F-tests and Pearson’s chi-square tests. Statistical significance was held at *p*-value< 0.05. All analyses were conducted using Stata/SE 15.1 [44].

## Results

### Retention strategies: successes and challenges

Routine weekly SAPPHIRE team meetings provided an outlet for field staff to discuss the successes and challenges of follow-up data collection with study management. With this information, study management could alter staff makeup, protocols, and the allocation of resources toward beneficial methods of follow-up. Key insights from these retention strategies are presented below.

#### Information obtained from locator forms

Detailed and accurate locator forms were essential for successful completion of follow-up visits. In addition to participant name and birthdate, the most beneficial pieces of information included: primary phone number(s); participant physical description; email address; social media accounts; and phone numbers and addresses of stable contacts. Detailed physical descriptions of participants helped field team members in identifying participants during data collection. Contacting participants through primary phone numbers emerged as a low-cost method of communicating with a large portion of participants. However, the most hard-to-reach participants often cycled through phone numbers or relied on “pay-as-you-go” cellphones that expire without payment.

Email and social media also served as critical no cost resources that improved the likelihood of locating a participant with minimal staff effort. These communication platforms are accessible on a variety of devices and allowed participants to engage with study staff whenever they could access their accounts. Participants with limited phone access, unreliable internet capability, and those with the propensity to change cell phones could and often did contact study staff using social media. Participants frequently visited fast food establishments with free Wi-Fi or hotels and libraries with computers to check their online accounts for messages. One of the greatest benefits of social media and email communication was that conversation histories were retained irrespective of duration since last contact or device used. Participants could see prior messages from study staff regardless of the time since the original contact attempts. This feature also allowed study staff to review prior conversations, setup subsequent interview sessions, and update locator information in REDCap based on past contact. Furthermore, social media photos supported pre-existing physical descriptions recorded on locator forms, which allowed study staff to more easily identify participants during data collection.

One challenge of using social media to locate participants was the occasional difficulty in locating accounts due to duplicate profiles or profiles created using a different name. Additionally, messages sent to study participants occasionally went to spam or junk folders and never reached participants. To minimize these issues, interviewers confirmed the correct account(s) during each study visit and sent a friend request with the participant’s permission. Once friend requests were accepted, messages went to the participant’s direct message folder and notified the participant.

When participants could not be reached directly, stable contacts provided information regarding participant whereabouts and updated phone numbers and addresses. Many participants listed parents, relatives, or romantic partners as stable contacts, some of which proved to be more useful than others. When making outreach calls or home visits, it was not uncommon to learn that the stable contact listed had not seen or communicated with the participant for an extended period of time. While unavoidable, study staff would document the finding in the participant’s REDCap file so that a new stable contact could be obtained during subsequent interactions. Once this issue became apparent, interviewers also encouraged participants to list other women enrolled in the study as stable contacts to create a network of women who were able to convey messages and locate each other for follow-up visits.

The one item on the locator form that was not useful for retention was the list of three locations frequented by participants. In practice, most participants listed the same convenience stores or prominent sex work areas within a recruitment zone. The likelihood of encountering a participant at one of these convenience stores was minimal, and staff were already spending time in these areas during van and tracking shifts.

#### Scheduling participants

Study management implemented the use of a participant database in REDCap after the start of 6-month follow-up interviews that allowed all staff to access locator forms, determine participant eligibility, and view previous contact attempts. REDCap also improved communication between field staff and reduced the time spent calling or visiting non-viable contacts. REDCap allowed study staff to remove incorrect participant information efficiently. Having a central participant database also allowed study management to audit participants contact history to ensure all possible methods had been attempted.

#### Use of a mobile van

Branded with the study logo, the study RV was recognizable and quickly became well-known among our target population. CFSW with no viable contact information frequented the van for outreach materials, to inquire about follow-up visits, and to seek refuge from inclement weather. The van provided a safe and private space for staff to speak with participants, update locator information, and complete follow-up interviews. In addition to being recognizable, the study van could accommodate simultaneous interviews, affording staff the capacity to complete up to eight interviews during a typical four-hour data collection shift.

There were also several disadvantages to using the van as a follow-up resource. The van’s large size made it difficult for study staff to drive and park throughout the city when conducting home visits, thus rendering its use for participant tracking negligible. Van shifts also required significant staffing resources. Due to the interview capacity of the van, the unpredictability of the number of interviews per shift, and the need for staff to sometimes canvas areas on foot, three staff members were needed during all van shifts. For many shifts, staff costs were incurred even though no interviews were obtained. Additionally, due to our targeted sampling recruitment strategy, dozens of participants were simultaneously eligible for follow-up visits in varying zones. As a result, van shift times and locations constantly varied each week. The unpredictability of the van shift schedule made it difficult for participants to know when the van would be in their area.

#### Participant tracking

Individualized participant tracking was employed to locate the study’s hardest-to-reach participants. The mobility of tracking teams and their focus on a select number of participants proved crucial to maintaining high retention. Tracking staff found participants during non-traditional hours and completed visits at convenient times for participants. Tracking teams frequently encountered potentially eligible women while conducting targeted outreach, who were approached and screened for study participation – enhancing the use of the extensive time spent on tracking. In general, tracking staff covered significantly more area than the study van and drastically increased the likelihood of random participant encounters. These staff members engaged with several peers, family members, and friends which helped establish rapport with the participant’s social network and ultimately, with the participant.

The primary drawback to participant tracking was the reliance on staff’s personal vehicles. In addition to placing an added burden on staff, the vehicles used were usually sedans or small vehicles and not physically designed for data collection. At times, this lack of space made interview administration difficult. Tracking interviews in personal vehicles also required participants to find private locations to collect vaginal swabs since there was no available restroom.

#### Maryland judiciary case search

Case Search emerged as a retention strategy that complemented the use of locator forms and individualized retention methods such as participant tracking and outreach. Occasionally, addresses listed in the locator form were incorrect from data entry or participant errors (e.g., missing apartment number, incorrect house number). By using publicly-listed case information, study staff were able to verify participant information and update errors in REDCap. At times, additional addresses were listed that study staff could visit to inquire about a participant’s location. Case search was also beneficial as it provided participant incarceration status, pending court cases, and sentence duration. After verifying a participant’s incarceration status, staff avoided wasting resources by not having to conduct home visits or phone calls to reach participants or their stable contacts.

There were several drawbacks to using Case Search. Since participants were not required to provide identification to enroll in SAPPHIRE, staff were limited to searching Case Search with reported names; thus, case information listed under different names or differently spelled names could be missed. To help mitigate this issue, study staff searched using variations of participant’s first and last names and birthdate. Additionally, entry of information into case search was not always entered in real time, resulting in outdated information. Information regarding case status and dispositions are also abbreviated and lack detail, so a participant’s current incarceration status was not always apparent.

#### Incentives

The $45 USD and $70 USD prepaid VISA debit cards greatly incentivized participants to return for follow-up visits. However, feedback from study participants indicated that cash could not be withdrawn from the prepaid debit cards, reducing the overall value of the incentive. Ultimately, SAPPHIRE study management chose not provide cash incentives due to quality assurance and staff safety.

The use of non-monetary incentives was also extremely beneficial for retention. Participants frequently stopped by the van or approached tracking teams to obtain items, thus increasing the likelihood of random encounters. For participants between visits, this provided the opportunity to distribute items branded with the study logo and phone number; participants used these items to call study staff and inquire about eligibility. Non-monetary incentives also helped with rapport building by providing an additional reason to interact women other than to inquire about eligibility.

While the non-monetary incentives provided during data collection were beneficial to sex workers, our sample was also characterized by high rates of drug use. Although we did distribute naloxone, retention efforts could have been further supported by providing additional harm reduction supplies such as safe injection and smoking materials (e.g., cookers, cotton, sterile water, stems), while also improving participant wellbeing.

#### Staff composition & rapport building

The cultural competency and diverse makeup of our staff was a tremendous asset to building rapport with our study population. Throughout the study, staff established and maintained relationships with participants through repeated positive encounters. It was common for participants to come to the study van or approach tracking teams and ask for staff members by name. Participants exemplified their comfort with our research team by providing unprompted information about peers that were also enrolled in the study (e.g., participant in treatment, jail, moved away), or giving study contact numbers to friends who had misplaced the information.

The greatest lesson learned regarding staff structure was the reliance on full-time staff versus the larger cadre of part-time staff and students. At the height of data collection, there were five distinct study visits occurring simultaneously, and it became evident that a full-time staff member specifically dedicated to retention was necessary. While casual staff and students served as low-cost data collectors, inconsistent availability and competing priorities restricted their ability to take ownership of participant retention. As a result, a full-time research assistant (RA) was hired to oversee study follow-up. This individual was tasked with assigning specific participants to field tracking teams and operating study phones and social media accounts. When participants became eligible, the full-time RA efficiently scheduled visits, deployed tracking teams, and audited outreach attempts to ensure exhaustion of contact methods before a participant’s eligibility window ended.

It is also possible that SAPPHIRE retention efforts could have benefited from the use of a peer navigator to assist with locating participants. Although a peer navigator was used with the SAPPHIRE study TFSW cohort not reported in this analysis, the use of peer navigators for the CFSW cohort would have required extensive effort and resources that were beyond our scope. Through participant interaction, it became apparent that familiarity among cisgender participants was primarily at the neighborhood level as opposed to citywide. Cisgender SAPPHIRE participants overwhelmingly stayed in the zones in which they were recruited. For peer navigators to be beneficial, the study would have needed multiple people familiar with each respective recruitment zone. Additionally, prior to the start of data collection, study management lacked rapport with women in our recruitment zones. Given the vulnerabilities experienced by our population, study management decided against the use of a peer navigator for retention to avoid creating a problematic dynamic in which an individual received financial incentives for locating peers within their network.

### Follow-up rates

**Fig. 1:**
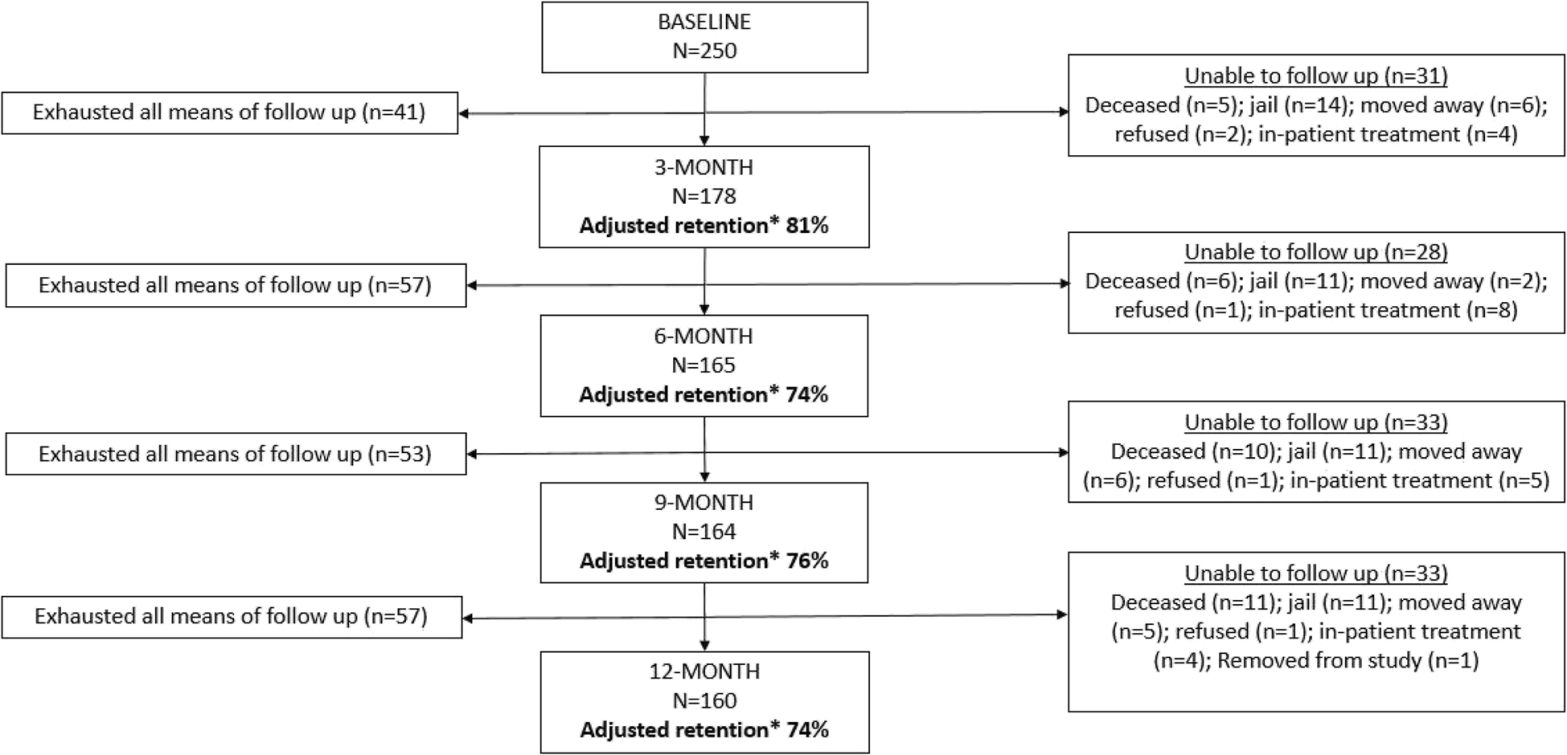
SAPPHIRE Study Participant Retention Flow

Of the original 250 individuals recruited, 178 (71%) completed the 3-month follow-up visit (Fig. 1). Of the 72 participants that were not retained during this interval, study staff exhausted all means of contact for 41 participants. The other 31 participants were unable to be contacted due to circumstances which prevented them from being followed, including being deceased, in jail, moving away, enrolled in in-patient drug treatment, or refusing to participate in follow-up. These individuals were removed from the total denominator given the inability to follow them, resulting in an adjusted retention proportion of 81%.

From the 3-month to 6-month follow-up, one participant who had previously refused to participate decided to re-engage. Twenty-eight participants were unable to be contacted for follow-up, and study staff exhausted all means of follow-up for 57, resulting in an adjusted 6-month retention proportion of 74%. From the 6- to 9-month follow-up, 33 participants were unable to be contacted for follow-up, and staff exhausted all means for 53 participants, resulting in adjusted 9-month retention of 76%. Between the 9-month follow-up and the final survey at 12 months, 33 participants were unable to be contacted, one of whom was removed from the study due to her conduct with study staff resulting in her being unable to complete the 12-month survey (all other data from this individual was included in analysis). Staff exhausted all means of follow-up for 57 participants. The adjusted 12-month retention was 74%.

Of the original 250 CFSW recruited, 41% completed all time points, 19% completed four time points, 18% completed three, 8% completed two, and 14% only completed baseline (Table 1). In comparing the number of visits (1–5) completed, women significantly differed in age at enrollment, relationship status, homelessness in the past 3-months, finding clients via referrals, and daily injection drug use. Women who only completed baseline were more likely to inject drugs daily at baseline as compared to women who completed more than one visit, and women who completed all 5 study visits were significantly less likely to experience homelessness in the past 3-months at baseline. There were no differences in racial/ethnicity composition, educational attainment, arrest, childhood abuse, daily engagement in sex work, number of clients, or time in street-based sex work.

**Table 1:** Baseline characteristics of SAPPHIRE participants by number of completed visits, N (%)

	Total	Baseline only	2 visits	3 visits	4 visits	5 visits	χ^2^ *p*-value
Characteristic	*n* = 250	*n* = 34	*n* = 19	*n* = 46	*n* = 48	*n* = 103	
Age, mean (SD)	35.7 (9.0)	33.3 (7.8)	31.7 (8.0)	37.1 (10.1)	32.6 (8.2)	37.9 (8.7)	**< 0.001**^a^
Race/ethnicity							0.133
White, non-Hispanic	166 (66.4)	24 (70.6)	13 (68.4)	26 (56.5)	27 (56.3)	76 (73.8)	
Black, non-Hispanic	57 (22.8)	6 (17.6)	4 (21.1)	16 (34.8)	11 (22.9)	20 (19.4)	
Hispanic or other	27 (10.8)	4 (11.8)	2 (10.5)	4 (8.7)	10 (20.8)	7 (6.8)	
Relationship status							**0.050**
In a relationship/married	84 (33.6)	8 (23.5)	3 (15.8)	17 (37.0)	12 (25.0)	44 (42.7)	
Single	165 (66.0)	26 (76.5)	15 (78.9)	29 (63.0)	36 (75.0)	59 (57.3)	
≥ 1 financial dependents	95 (38.0)	10 (29.4)	10 (52.6)	16 (34.8)	24 (50.0)	35 (34.0)	0.157
Children < 18 living with them	44 (17.6)	1 (2.9)	2 (10.5)	9 (19.6)	10 (20.8)	22 (21.4)	0.127
Less than high school/GED	131 (52.4)	18 (52.9)	13 (68.4)	20 (43.5)	24 (50.0)	56 (54.4)	0.448
Homeless, past 3 months	156 (62.4)	24 (70.6)	17 (89.5)	38 (82.6)	36 (75.0)	41 (39.8)	**< 0.001**
Food insecurity, past 3 month	135 (54.0)	17 (50.0)	12 (63.2)	29 (63.0)	29 (60.4)	48 (46.6)	0.245
Arrested, past 12 months	116 (46.6)	18 (52.9)	9 (47.4)	25 (54.3)	25 (53.2)	39 (37.9)	0.227
Experienced childhood abuse	126 (52.5)	21 (63.6)	9 (50.0)	20 (45.5)	23 (48.9)	53 (54.1)	0.575
Daily sex work	165 (66.0)	26 (76.5)	11 (57.9)	33 (71.7)	35 (72.9)	60 (58.3)	0.154
30+ clients in past 3 months	111 (44.8)	11 (32.4)	9 (47.4)	21 (45.7)	17 (35.4)	53 (52.5)	0.177
Ever find clients indoors	120 (48.4)	16 (47.1)	9 (47.4)	21 (46.7)	24 (51.1)	50 (48.5)	0.994
Ever find clients online	69 (27.8)	14 (41.2)	7 (36.8)	14 (31.1)	15 (31.9)	19 (18.4)	0.063
Ever find clients from referrals	110 (44.4)	11 (32.4)	8 (42.1)	24 (53.3)	14 (29.8)	53 (51.5)	**0.047**
Time in street-based sex work							0.065
6+ years	129 (51.6)	16 (47.1)	5 (26.3)	21 (45.7)	25 (52.1)	62 (60.2)	
≤ 5 years	121 (48.4)	18 (52.9)	14 (73.7)	25 (54.3)	23 (47.9)	41 (39.8)	
Daily non-injection drug use^b^	186 (74.4)	28 (82.4)	10 (52.6)	36 (78.3)	35 (72.9)	77 (74.8)	0.179
Daily injection drug use	146 (58.4)	28 (82.4)	11 (57.9)	26 (56.5)	28 (58.3)	53 (51.5)	**0.038**

^a^F-test ^b^excluding marijuana use

## Discussion

The SAPPHIRE study was one of the first cohort studies of street-based CFSWs in the U.S. The number of structural vulnerabilities (e.g., homelessness, frequent arrests) that characterized study participants required significant effort to ensure adequate retention. Overall, adjusted retention exceeded 70% for the duration of the 12-month study and 86% of participants completed at least one follow-up visit. These findings are comparable to other studies of FSW. For example, a recent study of drug involved FSW in Baltimore obtained 65% retention at 12 weeks [38]. In a study of FSW in Mexico, 82% of participants were retained for a 6-month follow-up interview [45]. In addition to studies of FSW, retention proportions mirror recent studies of similarly hard-to-reach populations. Among people experiencing homelessness, Fuehrlein et al. [46] retained 72% of participants over 2 years, and Caton et al. [47] were able to locate 85% of participants for at least one follow-up in an 18-month window. In a study of formerly incarcerated men, Fahmy et al. [48] retained 66% of participants one-month post release from prison, and 64% at 10-months.

Successful retention of CFSWs enrolled in the SAPPHIRE study was bolstered by a variety of retention strategies: collecting detailed locator information; outreach through social media and email; pre-scheduled van shifts; individualized participant tracking; public record searches; cash and non-cash incentives; and staff makeup and rapport building. Like other retention studies of hard-to-reach populations, there was no singular method that proved most effective when locating or maintaining contact with SAPPHIRE participants [9, 22, 25, 26]. Alternatively, several different strategies and techniques to enhance retention were used concurrently. By using multiple methods, the likelihood of locating participants greatly increased. Use of concurrent strategies also allowed study staff to obtain information about a participant’s whereabouts and then confirm the information through a second source.

Despite extensive effort to retain participants in the SAPPHIRE study, there were several women who could not be located. The most common reason for missing a follow-up interview was exhausting all means of contact (57–67% across all time points). When re-engaging with participants during future visits or random encounters, women often indicated that they had lost their phone, had it stolen, or ran out of minutes on prepaid phones and could not make or receive calls. Retention of CFSW may be enhanced by providing mobile phones or minutes that can be used with pay-as-you-go-phones.

A large portion of participants also missed visits due to reasons that prevented them from interacting with study staff. Between 12 and 19% of participants across all time points missed visits due to being incarcerated during their eligibility window. Given the high rates of incarceration among our sample and U.S. street-based CFSW more broadly [2, 49], future longitudinal studies of CFSW should consider developing protocols to be able to complete study visits in correctional facilities. Across all time points, 2–8% of participants missed study visits due to moving at least 1 h from Baltimore. Although phone interviews were permitted, study staff were still unable to obtain data for these participants. Protocols for telephone interviews should be incorporated into study design, and the opportunity to complete phone interviews should be clearly articulated to study participants. Lastly, 4–9% of participants missed visits while enrolled in-patient treatment. Participants enrolled in in-patient treatment are often unable to complete study visits due to “blackout” policies that prohibit them from communicating with anyone outside of the treatment facility. While missed visits by participants who are in treatment, incarcerated, or who have relocated are unavoidable, we strongly encourage detailed record keeping and the use of a digital database such as REDCap to monitor participant progress through the study to ensure successful study re-entry at future follow-up visits [25].

When examining demographic characteristics, retention differed by age at baseline, homelessness, relationship status, and daily injection drug use. Participants who were younger, recently experienced homelessness, and injected drugs daily were found to be less likely to have completed all or most follow-up visits. This finding supports previous research with FSW and other hard-to-reach populations [10, 11], underscoring the role of structural vulnerabilities in the ability to reliably locate participants over time.

Whereas older and more stably housed women may have been easily located through home visits and direct forms of contact such as phone calls or texts, retention of younger, more transient participants with a higher frequency of injection drug use may be bolstered through a greater emphasis on email or social media outreach that can be viewed on any device, or strategies that increase the likelihood of random encounters such as providing targeted non-cash incentives, or spending additional time in a recruitment area via tracking shifts or pre-scheduled van shifts. Although study staff distributed lip balm, hand sanitizer, sanitary wipes, and Naloxone, more unstable participants with higher rates of drug use may have been further incentivized to visit the study van by providing safe injection kits, fentanyl testing strips, safe crack cocaine smoking kits, or other harm reduction tools tailored to our target population. Additionally, while we were able to increase our field presence during times of heightened follow-up eligibility, the randomized recruitment strategy used resulted in a varying number of participants being eligible simultaneously throughout the city, and thus required a constantly evolving schedule. Alternatively, use of a fixed schedule may increase retention for field-based studies.

### Limitations

This research is characterized by several limitations. We did not systematically record the successful method of location for each follow-up interview completed. Alternatively, benefits and drawbacks of each method described in this analysis were derived from staff feedback during team meetings as opposed to systematically documented successes and failures. Future studies should build retention information into data collection systems. By recording successful methods of contact, study management can audit aggregate data to efficiently allocate resources and staff to retention strategies most beneficial for the population being studied.

A second limitation of our findings is the lack of verified information regarding participants who missed study visits due to in-patient treatment or relocation from Baltimore. In the event we were unable to reach participants, we used information from Maryland Judiciary Case Search, stable contacts, or unprompted information from other participants to determine if a participant was in treatment or relocated. When possible, however, the information was verified with the participant at subsequent follow-ups. Developing a protocol for accessing information from, and coordinating with treatment centers would be beneficial for verifying information and retaining additional participants.

Lastly, the demographic composition of our sample may prevent generalizability to other street-based sex worker populations. Although the SAPPHIRE sampling frame was developed using a plethora of sources (e.g., 911 calls for service, arrest data, and key informant interviews) [35], the cohort was 66% White whereas Baltimore City overall is 63% Black [50]. One possible explanation for the disproportionate sampling of white participants could be the preference of minority women to engage in sex work at indoor venues including exotic dance clubs and private residences to avoid arrest and police harassment [32, 51].

## Conclusion

Sex workers, PWUD, and people experiencing homelessness disproportionately experience negative health outcomes at higher rates than the general public, yet they are often absent from public health research and surveillance as a result of the difficulty and high costs of engagement and retention [9–12]. While researchers have examined barriers and facilitators to the retention of hard-to-reach populations, studies primarily examine the retention of samples recruited from fixed-sites or include FSW populations that are often broadly defined or do not differentiate between indoor or street-based venues of employment. Although there were drawbacks to each retention strategy, we found each method to be useful for the retention of SAPPHIRE study participants. However, overall stability of participants differed widely among the cohort, and retention strategies must be tailored based on participant characteristics. More stable participants appear to benefit from direct forms of contact (e.g., phone calls, social media, email). Alternatively, less stable participants require extensive field-based efforts such as home visits and tracking. By monitoring sample characteristics, study management can ensure there are adequate staff and resources to focus on strategies most likely to result in successful retention.

## Data Availability

Data unavailable due to privacy concerns for participants.

## Abbreviations

CAB: Community advisory board
CAPI: Computer Assisted Personal Interview
CFSW: Cisgender female sex worker
FSW: Female sex worker
PWUD: People who use drugs
RA: Research Assistant
REDCap: Research Electronic Data Capture
SAPPHIRE: Sex Workers and Police Promoting Health in Risky Environments
SSP: Syringe Services Program
TFSW: Transgender female sex worker

## References

[1] Campbell R, Kinnell H (2000). “We Shouldn’t have to put up with this”: street sex work and violence. Criminal Justice Matters.

[2] Footer KHA, Park JN, Allen ST, Decker MR, Silberzahn BE, Huettner S (2019). Police-related correlates of client-perpetrated violence among female sex Workers in Baltimore City, Maryland. Am J Public Health.

[3] Deering KN, Amin A, Shoveller J, Nesbitt A, Garcia-Moreno C, Duff P (2014). A systematic review of the correlates of violence against sex workers. Am J Public Health.

[4] Ciccarone D (2017). Fentanyl in the US heroin supply: a rapidly changing risk environment. Int J Drug Policy.

[5] Galea S, Vlahov D (2002). Social determinants and the health of drug users: socioeconomic status, homelessness, and incarceration. Public Health Rep.

[6] Hibbs JR, Benner L, Klugman L, Spencer R, Macchia I, Mellinger AK (1994). Mortality in a cohort of homeless adults in Philadelphia. N Engl J Med.

[7] Van Handel MM, Rose CE, Hallisey EJ, Kolling JL, Zibbell JE, Lewis B (2016). County-level vulnerability assessment for rapid dissemination of HIV or HCV infections among persons who inject drugs, United States. J Acquir Immune Defic Syndr.

[8] Zibbell JE, Iqbal K, Patel RC, Suryaprasad A, Sanders KJ, Moore-Moravian L (2015). Increases in hepatitis C virus infection related to injection drug use among persons aged ≤30 years - Kentucky, Tennessee, Virginia, and West Virginia, 2006-2012. MMWR Morb Mortal Wkly Rep.

[9] Bonevski B, Randell M, Paul C, Chapman K, Twyman L, Bryant J (2014). Reaching the hard-to-reach: a systematic review of strategies for improving health and medical research with socially disadvantaged groups. BMC Med Res Methodol.

[10] Sydor A (2013). Conducting research into hidden or hard-to-reach populations. Nurse Res.

[11] Lambert EY, Weibel WW The collection and interpretation of data from hidden populations. National Institute on Drug Abuse 1990. https://archives.drugabuse.gov/sites/default/files/monograph98.pdf. Accessed 12 Aug 2019.

[12] Abad N, Baack BN, O’Leary A, Mizuno Y, Herbst JH, Lyles CM. A systematic review of HIV and STI behavior change interventions for female sex workers in the United States. AIDS Behav. 2015;19:1701–19. 10.1007/s10461-015-1013-2 25711295

[13] Center for Disease Control and Prevention. HIV risk among adult sex workers in the United States. 2019. https://www.cdc.gov/hiv/group/sexworkers.html Accessed 12 Aug 2019.

[14] Cronbach LJ (1980). Toward reform of program evaluation.

[15] Riecken HW, Boruch RF. Social experimentation: a method for planning and evaluating social intervention. New York: Academic Press; 1974.

[16] Furimsky I, Cheung AH, Dewa CS, Zipursky RB (2008). Strategies to enhance patient recruitment and retention in research involving patients with a first episode of mental illness. Contemp Clin Trials.

[17] Lindenberg CS, Solorzano RM, Vilaro FM, Westbrook LO (2001). Challenges and strategies for conducting intervention research with culturally diverse populations. J Transcult Nurs.

[18] Brown-Peterside P, Rivera E, Lucy D, Slaughter I, Ren L, Chiasson MA (2001). Retaining hard-to-reach women in HIV prevention and vaccine trials: project ACHIEVE. Am J Public Health.

[19] Burns D, Soward ACM, Skelly AH, Leeman J, Carlson J (2008). Effective recruitment and retention strategies for older members of rural minorities. Diabetes Educ.

[20] Maher JE, Pranian K, Drach L, Rumptz M, Casciato C, Guernsey J (2010). Using text messaging to contact difficult-to-reach study participants. Am J Public Health.

[21] Hough RL, Tarke H, Renker V, Shields P, Glatstein J (1996). Recruitment and retention of homeless mentally ill participants in research. J Consult Clin Psychol.

[22] McCuller WJ, Sussman S, Holiday K, Craig S, Dent CW (2002). Tracking procedures for locating high-risk youth. Eval Health Prof.

[23] Morse EV, Simon PM, Besch CL, Walker J (1995). Issues of recruitment, retention, and compliance in community-based clinical trials with traditionally underserved populations. Appl Nurs Res.

[24] Festinger DS, Marlowe DB, Dugosh KL, Croft JR, Arabia PL (2008). Higher magnitude cash payments improve research follow-up rates without increasing drug use or perceived coercion. Drug Alcohol Depend.

[25] El-Khorazaty MN, Johnson AA, Kiely M, El-Mohandes AAE, Subramanian S, Laryea HA (2007). Recruitment and retention of low-income minority women in a behavioral intervention to reduce smoking, depression, and intimate partner violence during pregnancy. BMC Public Health.

[26] Escobar-Chaves SL, Tortolero SR, Mâsse LC, Watson KB, Fulton JE (2002). Recruiting and retaining minority women: findings from the women on the move study. Ethn Dis.

[27] Yancey AK, Ortega AN, Kumanyika SK (2006). Effective recruitment and retention of minority research participants. Annu Rev Public Health.

[28] Ashing-Giwa K, Rosales M (2012). Recruitment and retention strategies of African American and Latina American breast cancer survivors in a longitudinal psycho-oncology study. Oncol Nurs Forum.

[29] Rothschild SK, Martin MA, Swider SM, Lynas CT, Avery EF, Janssen I (2012). The Mexican-American trial of community health workers (MATCH): design and baseline characteristics of a randomized controlled trial testing a culturally tailored community diabetes self-management intervention. Contemporary Clinical Trials.

[30] Beyrer C, Crago A-L, Bekker L-G, Butler J, Shannon K, Kerrigan D (2015). An action agenda for HIV and sex workers. Lancet..

[31] Sherman SG, Footer K, Illangasekare S, Clark E, Pearson E, Decker MR (2015). “What makes you think you have special privileges because you are a police officer?” a qualitative exploration of police’s role in the risk environment of female sex workers. AIDS Care.

[32] Sherman SG, Park JN, Galai N, Allen ST, Huettner SS, Silberzahn BE (2019). Drivers of HIV infection among Cisgender and transgender female sex worker populations in Baltimore City: results from the SAPPHIRE study. J Acquir Immune Defic Syndr.

[33] El-Bassel N, Witte SS, Wada T, Gilbert L, Wallace J (2001). Correlates of partner violence among female street-based sex workers: substance abuse, history of childhood abuse, and HIV risks. AIDS Patient Care STDs.

[34] Inciardi JA, Surratt HL, Kurtz SP (2006). HIV, HBV, and HCV infections among drug-involved, inner-city, street sex workers in Miami, Florida. AIDS Behav.

[35] Allen ST, Footer KHA, Galai N, Park JN, Silberzahn B, Sherman SG (2018). Implementing targeted sampling: lessons learned from recruiting female sex Workers in Baltimore, MD. J Urban Health.

[36] Tomko C, Park JN, Allen ST, Glick J, Galai N, Decker MR (2019). Awareness and interest in HIV pre-exposure prophylaxis among street-based female sex workers: results from a US context. AIDS Patient Care STDs.

[37] Park JN, Footer KHA, Decker MR, Tomko C, Allen ST, Galai N (2019). Interpersonal and structural factors associated with receptive syringe-sharing among a prospective cohort of female sex workers who inject drugs: receptive syringe sharing among FSW-IDU. Addiction..

[38] Decker MR, Tomko C, Wingo E, Sawyer A, Peitzmeier S, Glass N (2017). A brief, trauma-informed intervention increases safety behavior and reduces HIV risk for drug-involved women who trade sex. BMC Public Health.

[39] Decker MR, Pearson E, Illangasekare SL, Clark E, Sherman SG (2013). Violence against women in sex work and HIV risk implications differ qualitatively by perpetrator. BMC Public Health.

[40] Sherman SG, Lilleston P, Reuben J (2011). More than a dance: the production of sexual health risk in the exotic dance clubs in Baltimore, USA. Soc Sci Med.

[41] Harris PA, Taylor R, Thielke R, Payne J, Gonzalez N, Conde JG (2009). Research electronic data capture (REDCap)--a metadata-driven methodology and workflow process for providing translational research informatics support. J Biomed Inform.

[42] Harris PA, Taylor R, Minor BL, Elliott V, Fernandez M, O’Neal L (2019). The REDCap consortium: Building an international community of software platform partners. J Biomed Inform..

[43] Maryland Judiciary Case Search [Internet]. [cited 2019 Jun 12]. Available from: http://casesearch.courts.state.md.us/casesearch/.

[44] StataCorp (2019). Stata statistical software: release 16.

[45] Patterson TL, Semple SJ, Fraga M, Bucardo J, De la Torre A, Salazar-Reyna J (2006). A sexual risk reduction intervention for female sex workers in Mexico: design and baseline characteristics. J HIV AIDS Soc Serv.

[46] Fuehrlein BS, Cowell AJ, Pollio D, Cupps L, Balfour ME, North CS (2015). A prospective study of the associations among housing status and costs of services in a homeless population. Psychiatr Serv.

[47] Caton CLM, Dominguez B, Schanzer B, Hasin DS, Shrout PE, Felix A (2005). Risk factors for long-term homelessness: findings from a longitudinal study of first-time homeless single adults. Am J Public Health.

[48] Fahmy C, Clark KJ, Mitchell MM, Decker SH, Pyrooz DC. Method to the madness: tracking and interviewing respondents in a longitudinal study of prisoner reentry. Sociol Methods Res. 2019;0049124119875962.

[49] Footer KH, Silberzahn BE, Tormohlen KN, Sherman SG (2016). Policing practices as a structural determinant for HIV among sex workers: a systematic review of empirical findings. J Int AIDS Soc.

[50] U.S. Census Bureau QuickFacts: Baltimore city, Maryland (County) [Internet]. Census Bureau QuickFacts. United States Census Bureau; [cited 2019 Jun 14]. Available from: https://www.census.gov/quickfacts/fact/table/baltimorecitymarylandcounty/AGE295217.

[51] Brantley ML, Kerrigan D, German D, Lim S, Sherman SG (2017). Identifying patterns of social and economic hardship among structurally vulnerable women: a latent class analysis of HIV/STI risk. AIDS Behav.

